# Rebuttal to Correspondence on “Marine Scrubbers vs Low-Sulfur Fuels: A Comprehensive Well-To-Wake Life Cycle Assessment Supported by Measurements Aboard an Ocean-Going Vessel”

**DOI:** 10.1021/acs.est.6c06523

**Published:** 2026-07-30

**Authors:** Patritsia M. Stathatou, Ievgenii Petrunia, Torsten Barenthin, George Gotsis, Paul Jeffrey, Christopher Fee, Scott Bergeron, Marios Tsezos, Michael Triantafyllou, Neil Gershenfeld

**Affiliations:** † School of Chemical and Biomolecular Engineering, 1372 Georgia Institute of Technology, Atlanta, Georgia 30332, United States; ‡ Renewable Bioproducts Institute, 1372 Georgia Institute of Technology, Atlanta, Georgia 30332, United States; § Center for Bits and Atoms, 2167 Massachusetts Institute of Technology, Cambridge, Massachusetts 02139, United States; ∥ Oldendorff Carriers GmbH & Company KG, Willy-Brandt-Allee 6, 23554 Lübeck, Germany; ⊥ Naias Laboratories, S.A., Piraeus 185 40, Greece; # Laboratory of Environmental Science and Engineering, 414916 School of Mining and Metallurgical Engineering, National Technical University of Athens, Athens 15773, Greece; ∇ Department of Mechanical Engineering, 2167 Massachusetts Institute of Technology, Cambridge, Massachusetts 02139, United States

We thank the authors of the Correspondence (DOI: 10.1021/acs.est.5c15904) for their interest in our work and for raising important methodological LCA considerations. Below, we address each point in detail and clarify the rationale behind our choices. Our analysis follows established LCA standards, uses the best available background data, and is directly informed by onboard measurements. As clarified below, several of the concerns raised appear to result from misinterpretations of reported values, units, or methodological descriptions. We therefore maintain that our conclusions are appropriately scoped, transparent, and supported by the data and methodology presented. At the same time, we acknowledge that the marine eutrophication and marine ecotoxicity results are associated with substantial uncertainty due to limitations in current LCIA methodologies and characterization models. While HFO with a scrubber showed comparable or lower impacts than the low-sulfur fuels across most impact categories (7 out of 10), it did not outperform the alternatives in marine eutrophication and marine ecotoxicity, and MGO showed lower WtW PM impacts. As discussed in the original article, PM abatement options should be adopted, while the marine eutrophication and ecotoxicity results should be interpreted with caution. Our intention was not to dismiss these LCIA impact categories by pointing this, but rather to highlight the limitations of current LCIA methodologies for assessing marine ecosystem impacts from moving ocean-going vessels in open seas. Further work on improving marine eutrophication and ecotoxicity LCIA methodologies, together with additional measurement-informed studies, will be important for more robustly assessing marine ecosystem impacts within LCA, and answering this question with greater confidence.

## Interpretation of Results

### Critical Impact Categories Are Omitted

We respectfully disagree that marine eutrophication and marine ecotoxicity were omitted or dismissed. These impact categories were explicitly calculated, reported in Figure 5, and discussed in Section 3.3, where we clearly state that the calculated TtW marine eutrophication and ecotoxicity impacts of HFO with a scrubber are approximately 6 and 3 times higher, respectively, than those of the low-sulfur fuels. Our interpretation was not that these results should be ignored, and our caution is not based on a general statement that “there is uncertainty”. On the contrary, we emphasize that they should be interpreted with caution because the available LCIA characterization factors are not well suited to represent scrubber washwater discharges from a moving ocean-going vessel in open seas.

Specifically, the characterization factors used for these impact categories do not distinguish between stationary discharge sources, such as wastewater treatment plants, and moving vessels. This distinction is important because dilution and dispersion conditions are fundamentally different for a vessel moving in open sea, where propeller-induced turbulence, hull displacement, currents, and continuous movement lead to rapid dilution of the discharged washwater. As discussed in Section 3.3, assuming only a conservative initial dilution factor of 10 due to these reasons, the calculated TtW marine eutrophication and ecotoxicity impacts become lower than the corresponding WtT impacts, leading to similar WtW impacts among the three fuel systems. This example highlights the limitation of current LCIA methods, that do not adequately account for near-field dilution, dispersion, exposure duration, or the moving nature of the source. This limitation is particularly consequential for marine eutrophication and ecotoxicity because the calculated impacts are driven by washwater constituents released directly to seawater, for which fate, exposure, and concentration at the point of ecological relevance strongly determine the actual potential for harm.

For this reason, we complemented the LCIA results with a detailed concentration-based assessment in Section 3.2.3. Nutrient concentrations were compared with US and EU municipal wastewater discharge limits, while metals and organic compounds were compared with strict US and EU industrial discharge limits. In addition, their expected concentrations in receiving water bodies, were also compared, after a conservative 1000-fold open-sea dilution, with EU environmental quality standards and US EPA aquatic-life criteria. The measured maximum concentrations in scrubber washwater and the diluted receiving-water concentrations were well below the relevant limits and environmental criteria, in many cases by orders of magnitude.

Therefore, our conclusion is that the Figure 5 results are important for transparency and discussion, but cannot by themselves support the conclusion that HFO with a scrubber has greater actual marine eutrophication or ecotoxicity impacts than low-sulfur fuels under the specific conditions studied: large ocean-going vessels operating in open seas.

### Discriminating Ecotoxicity and Marine Eutrophication

The purpose of the 10-fold dilution example was not to modify characterization factors arbitrarily or to disregard the basis on which they are derived, but to illustrate the sensitivity of these two specific indicators to an important fate-process limitation: the analyzed source is a moving ocean-going vessel, not a stationary discharge point. This distinction is directly relevant for pollutants discharged to seawater, because near-field dilution, wake turbulence, propeller mixing, and vessel movement strongly affect exposure concentrations and residence times in the receiving environment. This is not analogous to CO_2_ emissions, for which climate change characterization factors are based on atmospheric persistence and globally integrated radiative forcing over a defined time horizon, not local receiving-environment exposure concentrations.^1,2^

Therefore, the dilution example was included to show that the Figure 5 marine eutrophication and ecotoxicity results are highly sensitive to dispersion assumptions that are not adequately captured by the applied LCIA characterization factors. This is consistent with our statement that these categories should be interpreted with caution rather than used alone to conclude greater actual marine ecosystem impacts from HFO with a scrubber in open-sea operation.

In addition, the LCIA method applied in our study (ReCiPe) represents an established and widely used approach within the LCA community. For marine eutrophication, the selected midpoint approach is consistent with the method recommended by the European Commission JRC/ILCD guidance,^3^ although it is still classified as requiring further improvement. For ecotoxicity, JRC explicitly states that no method is currently recommended for marine ecotoxicity, reflecting the limited maturity of available characterization models. To our knowledge, there is no approved or widely accepted marine ecotoxicity LCIA method that includes alkylated PAHs.

Regarding alkylated PAHs and other substances not included in the characterization method used, we agree that incomplete substance coverage is an important limitation of current LCIA methods and should be part of the broader discussion on improving marine ecotoxicity characterization. However, this limitation does not by itself demonstrate that marine ecotoxicological impacts are “likely higher.” In our measurements, parent PAHs were either not detected or detected at very low concentrations, total PAHs were far below IMO thresholds, BTEX compounds were largely not detected, 1,4-dichlorobenzene was not detected, and oil and grease were below 1 mg/L in both inlet and outlet samples. While alkylated PAHs were not directly measured, and therefore cannot be fully excluded, the very low concentrations of related organic indicators suggest that their presence at environmentally significant concentrations is unlikely under the conditions studied.

## Methodological Inconsistencies

### Selection of a Functional Unit

The functional unit used in our study is one MJ of energy input to the vessel engines (MJ_in_), not one MJ of fuel input to “the ship”. For the WtT assessment, fuel inventories were converted to this functional unit using each fuel’s calorific value. Scrubber production impacts were assessed both per scrubber produced and per MJ_in_ over the scrubber’s 20-year lifetime, based on the vessel's independently verified annual HFO consumption data provided by Oldendorff, in accordance with the regulatory requirements of the IMO Data Collection System (DCS). This annual fuel consumption reflects real operation of the HFO-scrubber system, including the additional fuel required to generate the auxiliary power needed for scrubber operation. This approach allowed scrubber production impacts to be expressed consistently with the fuel-related impacts and integrated into the WtW assessment.

The scrubber energy requirement is also included in the TtW analysis. Onboard emission factors were calculated from measured emissions, fuel consumption, and total engine output, including both the main engine and the auxiliary engines. In the HFO-scrubber case, the auxiliary engine load includes the electrical power required to operate the scrubber. Therefore, the additional fuel consumption and emissions associated with the approximately 80–100 kW scrubber power demand are not canceled out or discarded; they are part of the measured operating conditions and are carried through the conversion of emissions from g/kWh of engine output to g/MJ_in_. In this sense, the functional unit used in our study explicitly and quantitatively captures scrubber operation, rather than omitting it (the scrubber load is already embedded in the measured fuel consumption and auxiliary engine operation before the impacts are expressed per MJ_in_).

We agree that transport work-based functional units, such as ton-km or propeller shaft work, are appropriate for studies comparing transport efficiency or propulsion-system performance. However, they are not aligned with the objective of this study, which was to compare fuel/scrubber systems and their WtW impacts under measured onboard operating conditions. A propeller-work functional unit would directly represent main-engine propulsion output (only the main engine powers the propeller), but would not represent the scrubber’s function, which is to treat the exhaust generated from the total fuel burned (both main and the auxiliary engines are connected to the scrubber – see SI Note 5), while using auxiliary-engine power. The MJ_in_ functional unit therefore provides a consistent basis across WtT and TtW stages and more directly accounts for both propulsion-related fuel use and the additional energy required for scrubber operation.

These methodological choices are mentioned in Section 2.1 and explained in detail in the Supporting Information (SI Note 4) of the article.

### Analyses of Marine Ecotoxicity and Eutrophication

We thank the authors of the correspondence for identifying this typographical error, where some concentrations in Section 3.2.3.3 were reported in μm/L instead of the correct unit, μg/L. The values appear in the correct units in the SI. We have submitted an Addition and Correction so that this error can be corrected for the record.

However, this comment appears to have misread both the reported values and their context. The TtW marine ecotoxicity value reported in Figure 5 and Section 3.3 for HFO with scrubber is 0.7 ± 0.1 g 1,4-dichlorobenzene-Eq/MJ_in_, not 0.07, as the comment states. The 0.07 ± 0.01 g 1,4-dichlorobenzene-Eq/MJ_in_ value is explicitly presented only after applying the illustrative conservative 10-fold dilution factor (as stated in the relevant Section 3.3. “Assuming a conservative initial dilution factor_···_ from 0.7 ± 0.1 g 1,4-dichloronenzene-Eq/MJ_in_ to 0.07 ± 0.01 g 1,4-dichloronenzene-Eq/MJ_in_, respectively”). Also, this is a TtW washwater-emission result, not a scrubber-production impact over the 20-year lifetime; the latter applies only to the WtT assessment of scrubber manufacturing.

The marine ecotoxicity calculation included the measured washwater substances with available ReCiPe characterization factors, i.e., the following metals and organic compounds: 1,4-dichlorobenzene, Ni, Cr, Co, Cu, Pb, Hg, V, Zn, fluoranthene, fluorene, phenanthrene, hydrocarbons, and toluene. For each substance, the vessel-weighted average concentration in washwater (in μg/L) was converted to g/MJ_in_ using the vessel-weighted washwater discharge rate (in L/h), average engine output (in kWh_out_), engine efficiency, and relevant unit conversions from kWh to MJ. Thus, the scrubber discharge flow rate and the kWh-to-MJ conversion were already included in the calculation.

Finally, the SI comparison appears to have also been misquoted. The reported value is 0.07 ± 0.01 kg 1,4-dichlorobenzene-Eq/m^3^, not g/m^3^ as stated in the comment, and it corresponds to the undiluted measured washwater composition. This value is correct and was calculated directly from the vessel-weighted average concentration of each substance considered in the marine ecotoxicity indicator, by converting concentrations from μg/L to kg/m^3^ using the conversion 1 μg/L = 10^–6^ kg/m^3^, and then multiplying by the corresponding ReCiPe characterization factors. Therefore, the commenters’ recalculation claiming a 35-fold increase and the associated extrapolated damage-cost estimate is not supported. On the contrary, our value is more than 3 times lower than the one previously reported in the literature, i.e., 0.23 kg 1,4-DCB-Eq/m^3^,^4^ as stated in SI Note 15, and not in SI Note 14 or in g/m^3^ as stated in the comment.

### Different Engine Conditions

The study indeed considers four engine operating modes (idle, ∼ 25%, ∼ 50%, and ∼ 80% MCR), applied consistently across all three fuel/scrubber systems, following the ISO 8178 steady-state discrete-mode test cycle (E2).^5^ This approach is specifically designed to capture the full range of the vessel’s operating conditions. All TtW emissions (gaseous, PM, and washwater) were measured and reported per mode (Section 3.2, Figures 2–4) and then combined using a weighted-average approach, also prescribed by ISO 8178, to derive representative emission factors and enable consistent LCA calculations and comparisons. Therefore, the adopted methodology reduces, rather than introduces, uncertainty by systematically accounting for operational variability.

The weighting factors used to derive both the representative emission factors and the average engine efficiency (∼0.46) are based on actual operational data, not assumptions. As detailed in SI Note 7, these factors were determined from hourly engine output data (% MCR) for the Hedwig Oldendorff vessel over nearly two years (January 2022–October 2023). During this period, the engine operated at idle for 37% of the time, ≤ 35% load for ∼ 12%, 35–60% load for ∼ 43%, and >60% load for ∼ 8%. These data-driven weights were then applied consistently across all fuels and modes. This approach represents the vessel’s real operational profile as accurately as possible and is fully aligned with established ISO practices for emission factor determination.

### Selection of Background Inventory Data

Indeed, the selected ecoinvent data set is not an exact MGO data set; however, it is the closest available proxy for a low-sulfur petroleum distillate fuel, with a calorific value of 42.8 MJ/kg (as reported in the database), matching the measured MGO used in this study. Importantly, the data set does not represent fuel with 10 ppm sulfur. Instead, it is based on a refinery processing average European crude (∼1% S), where the necessary desulfurization steps to produce low-sulfur distillates are embedded in the refinery model. Therefore, it captures the relevant refining energy demand and associated emissions. There is no indication in the ecoinvent documentation (v3.10.1) that this data set represents 10 ppm automotive diesel or that it is based on Jungbluth et al. (2018).^6^ Rather, as mentioned in the documentation of the database, it relies on a BREF-calibrated refinery model (ifeu tool),^7^ representing European refinery operations and producing low-sulfur distillate fuels across multiple sectors, including automotive, railroad, aviation, agricultural, and military uses. Thus, it cannot correspond to a single fixed sulfur specification such as 10 ppm, but instead represents a generic low-sulfur distillate fuel.

To the best of our knowledge, this data set constitutes the most appropriate, trustworthy, and widely used proxy for MGO within ecoinvent to represent emissions from global petroleum fuel production. Using this data set results in a WtT carbon intensity of 0.94 kg CO_2_e/kg fuel for MGO, which corresponds to 22 g CO_2_e/MJin when accounting for its calorific value. The calculation methodology, including consistency across all fuels, is described in detail in SI Note 1. These values are reflected in the results presented in Figure 5 and Figure S16; there is no Figure 6 in the article, as referenced in the comment. The results consistently show approximately 24% lower WtT emissions for HFO + scrubber compared to MGO, based on the applied methodology. Finally, we verified these findings using the most recent ecoinvent version (v3.12), and the relative differences remain unchanged.

Therefore, the background data used in this study are appropriate, consistent with best available LCA practice, and suitable for the comparative analysis performed.
